# Global disease burden and its attributable risk factors of peripheral arterial disease

**DOI:** 10.1038/s41598-023-47028-5

**Published:** 2023-11-14

**Authors:** Yayu You, Zhuo Wang, Zhehui Yin, Qinyi Bao, Shuxin Lei, Jiaye Yu, Xiaojie Xie

**Affiliations:** 1 Department of Cardiology, Second Affiliated Hospital, Zhejiang University School of Medicine, 88 Jiefang Road, Hangzhou, 310009 Zhejiang China; 2 International Institutes of Medicine, Fourth Affiliated Hospital, Zhejiang University School of Medicine, Yiwu, 322000 China

**Keywords:** Risk factors, Peripheral vascular disease

## Abstract

Peripheral arterial disease (PAD) is a prevalent subtype of atherosclerotic cardiovascular diseases. It is crucial to assess the PAD-related burden and its attributable risk factors. We use the Global Burden of Disease study 2019 database to calculate the incidence, prevalence, mortality, disability-adjusted life years (DALY), attributable risk factors and estimated annual percentage change. The disease burden of PAD grows significantly with age accompanied by prominent heterogeneity between male and female. Despite the increase in the absolute numbers of disease burden from 1990 to 2019, the global PAD-related age-standardized death rate (ASDR) and age-standardized disability-adjusted life years rate (ASDALYR) have a mild downward trend from 1990 to 2019, which negatively correlated with sociodemographic index (SDI). Smoking and high systolic blood pressure (SBP) were the primary attributable risk factors for males (ASDR: 33.4%; ASDALYR: 43.4%) and females (ASDR: 25.3%; ASDALYR: 27.6%), respectively. High fasting plasma glucose (FPG) had become the second risk factor for ASDR (males: 28.5%; females: 25.2%) and ASDALYR (males: 29.3%; females: 26.3%) with an upward tendency. Low-middle SDI regions were predicted to have the most remarkable upward trend of PAD-related burden caused by high FPG. Smoking caused more disease burden in males before 85–90 years old and females before 65–70 years old, while high FPG and high SBP caused more burden after that. The patterns of PAD-related burden and its attributable risk factors are heterogeneous across ages, genders, and SDI regions. To reduce disease burden, tailored strategies should be implemented.

## Introduction

Peripheral artery disease (PAD) is a major atherosclerotic cardiovascular disease (ASCVD) that affects over 118.1 million people worldwide in 2017^1^. Patients with PAD have an extensive atherosclerotic burden and increased risk of major adverse cardiovascular events^2,3^. In recent decades, coronary heart disease and stroke prevention and burden management have improved. However, less attention has been paid to PAD by public health experts and policymakers than other ASCVDs^4^.

According to the Global Burden of Disease Study (GBD) 2017, the global number of years lived with disability (YLD) was 515.6 thousands in PAD, representing a significant public health concern^1^. A previous systematic review reported that 236.62 million (5.56%) individuals aged 25 years old (y/o) or older had PAD, with almost 70% in low- and middle-income countries, indicating a shifted epidemiological pattern. Many studies have shown that elderly, smoking, hypertension, diabetes, and hypercholesterolemia may contribute to PAD^5^. Despite past research on PAD epidemiology, much remains to be done. Compared to GBD 2017 studies, GBD 2019 used new methods to better measure attributable risk factors by integrating multiple high-quality epidemiologic studies^6^.

In our study, we analysed the updated GBD 2019 database on PAD prevalence, incidence, death, disability-adjusted life-years (DALYs), and risk factors in the global population, stratified by age, gender, sociodemographic index (SDI), countries, and territories. The estimated annual percentage changes (EAPCs) were utilized to assess temporal trends. Our study will help understand the current trend of PAD-related burden and its attributable risk factors, thereby facilitating specific response and health care policy.

## Method

### Data source

All data we utilized were collected by the GBD study and downloaded from Global Health Data Exchange(GHDx, http://ghdx.healthdata.org/gbd-results-tool), including PAD deaths, DALYs, Years of Life Lost (YLL), YLD, incidence, prevalence and their age-standardized rate(ASR), attributable risks factors in 204 counties from 1990 to 2019. The GBD study methodology has been introduced in detail previously^7^. Published studies, organization websites, and primary data sources provide vast amounts of disease data. PAD was identified by the International Classification of Diseases and Injuries version 10 (ICD-10) discharge diagnosis code. PAD corresponds to ICD-10 codes I70.2-I73.9. According to these data, Cause of Death Ensemble model (CODEm), DisMod-MR 2.1, and spatiotemporal Gaussian process regression (ST-GPR) were used^8–10^. Previous reports detail CODem and DisMod-MR 2.1. Furthermore, the GBD study divides the world into five parts based on SDI level. SDI, a composite indicator, is measured by a country's lag-distributed income per capita, average years of schooling, and the fertility rate in females under 25 y/o^11^. Also, income is calculated using the World Bank Atlas method to convert local currency to gross national income (GNI) per capita, in dollars^12^. World Bank income (WBI) levels divide the world into 4 regions: low, lower middle, upper middle, and high^13^. The GBD 2019 estimation of 87 risk factors followed the comparative risk assessment framework (CRA)^14^. In essence, CRA involves six primary steps: 1. incorporating risk-outcome pairs; 2. calculating relative risk; 3. estimating exposure and its distributions; 4. identifying the counterfactual level of exposure and the theoretical minimum risk exposure level (TMREL); 5. determining the population of attributable fraction (PAF) and the attributable burden; 6. computing the burden attributable to combinations of risks^6^. Through CRA, 6 attributed risks including high fasting plasma glucose (FPG), high systolic blood pressure (SBP), smoking, kidney dysfunction, diet high in sodium and lead exposure were obtained for PAD.

### Statistical analysis

Age-standardized death rate (ASDR), age-standardized disability-adjusted life years rate (ASDALYR), age-standardized years lived with disability rate (ASYLDR), age-standardized years of life lost rate (ASYLLR), age-standardized incidence rate (ASIR) and age-standardized prevalence rate (ASPR) were showed by per 100,000 population categorized by age, gender, geographical location and social development. From 1990 to 2019, percentage changes in cases or rates were defined as follows:

$$  {\text{Percentage change of cases }}\%  = 100\% {\text{*}}\frac{{\left( {{\text{Cases }}2019 - {\text{ Cases }}1990} \right)}}{{{\text{Cases }}1990}}  $$

$$ {\text{Percentage change of rate}}\,\%  = 100\% {\text{*}}\frac{{\left( {{\text{Rates }}2019 - {\text{ Rates }}1990} \right)}}{{{\text{Rates }}1990}} $$

To assess and predict the temporal trend of age-standardized rate (ASR), the estimated annual percent change (EAPC) and 95% confidence intervals (CI) were calculated using a Generalized Linear Model (GLM) for ASR during 30 years^15^. The formula of EAPC was showed followed:

$$ {\text{EAPC }} = { }100{ } \times { }\left( {{\text{exp}}\left( {\upbeta } \right){ } - { }1} \right) $$

where β was the annual change per 100,000 in the ASR. Positive EAPC was considered to increase ASR, while negative EAPC decreased ASR^16,17^. Regression Splines and a smoothed line were used to show the relationship between SDI and EACP in ggplot2 and R with “stat_smooth”. To display the proportion of PAD cases that could be prevented if a risk was removed, population attributable fraction (PAF) was calculated. The PAF calculation method was published. The formula for PAF was shown:

$$   {\text{PAF}} = \frac{{\text{A}}}{{\text{O}}} \times 100\%   $$

where A and O refer to attributable risk the observed number of cases and the expected number of cases under no exposure, respectively.

## Results

### Current burden of PAD

In 2019, there were 10.5 million (95% UI [9.2–12]) incident cases and 113.4 million (95% UI [99.2–128.4]) prevalent cases of PAD (Supplementary Table 1), with an ASIR of 127.11 (95% UI [111.28–145.44]) per 100,000 population and an ASPR of 1401.85 (95% UI [1228.48–1589.39] per 100,000 in 2019 (Supplementary Table 2). From 1990 to 2019, the global ASIR dropped by 18.91%. The largest drop was in high SDI regions (− 32.09%) and high WBI countries (− 31.52%). The global ASPR showed similar declining condition.

In 2019, PAD caused approximately 74.1 thousand deaths globally (95% UI [41.2–128.2]), which increased 1.47-fold from 30 thousand deaths (95% UI [16.2–52.4]) in 1990. In 2019, high and high-middle SDI regions contributed 82.3% of PAD-related deaths (Supplementary Table 1). Globally, the ASDR of PAD slightly dropped by 2.45% from 1990 to 2019, and the most prominent decrease of ASDR was observed by − 5.33% in high-middle SDI regions, and − 5.43% in upper-middle WBI countries. The largest increase was observed in low-middle SDI (55.44%) and low WBI regions (25.03%). (Supplementary Table 2).

In 2019, PAD caused 1.5 million (95% UI [1–2.4]) DALY globally, including 1 million YLL (95% UI [0.6–1.8]) and 0.5 million (95% UI [0.2–0.9]) YLD. High and high-middle SDI regions contributed 80.1% of PAD-related DALYs. The global ASDALYR dropped by 12.75% from 1990 to 2019. Similar to the ASDR, the greatest increases were observed in low-middle SDI regions (11.01%) and low WBI countries (12.1%). The greatest decreases were observed in high-middle SDI regions (-13.11%) and high-middle WBI countries (− 11.5%) (Supplementary Table 2).

Regionally, the largest ASIRs in 2019 were observed in high-income North America (193.5, 95% UI [173.75–215.57]) and Western Europe (165.21, 95% UI [143.28–189.11]). The lowest ASIRs were observed in both Andean Latin America (84.49, 95% UI [73.13–96.85]) and South Asia (93.07, 95% UI [80.54–106.50]). High-income North America (2214.33, 95% UI [1986.68–2433.81]) and Western Europe (1902.51, 95% UI [1659.40–2145.42]) had the highest ASPRs in 2019, while Andean Latin America (828.7, 95% UI [715.35–951.23]) and Western Sub-Saharan Africa (902.73, 95% UI [783.35–1036.44]) had the lowest ASPRs (Supplementary Table 2).

The highest ASDRs were observed in Eastern Europe (3.49, 95% UI [1.68–6.68]) followed by Australasia (2.47, 95% UI [1.19–4.70]). The lowest ASDRs were observed both in Andean Latin America (0.14, 95% UI [0.10–0.17]) and East Asia (0.14, 95% UI [0.11–0.18]). Eastern Europe (63.58, 95% UI [33.52–117.79]) and Southern Sub-Saharan Africa (41.79, 95% UI [33.36–49.85]) had the highest ASDALYRs in 2019, while Andean Latin America (5.85, 95% UI [3.69–9.18]) and high-income Asia Pacific (7.49, 95% UI [4.50–11.98]) had the lowest ASDALYRs (Supplementary Table 2).

**Figure 1:**
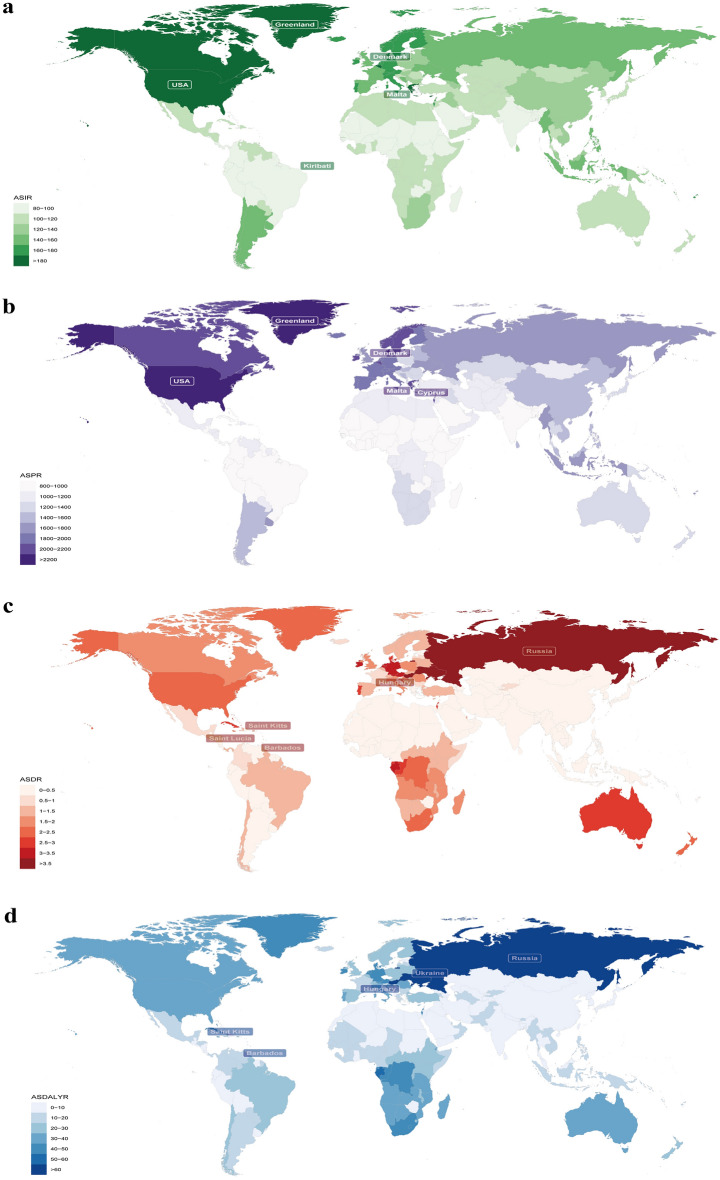
The global burden of peripheral arterial disease in 204 countries and territories. (**a**) The age-standardized incidence rate in 2019. (**b**) The age-standardized prevalence rate in 2019. (**c**) The age-standardized death rate in 2019. (**d**) The age-standardized DALYs rate in 2019. DALY: disability-adjusted life-year. Maps generation was performed using the open-source software R (version 4.1.0) with package of “maps” (URL of Package ‘maps’: https://cran.r-project.org/web/packages/maps/index.html; under a GNU general public license version 2: https://cran.r-project.org/web/licenses/GPL-2).

To elucidate the substantial between-country variations of ASIR, ASPR, ASDR, and ASRDALY of PAD, the global distribution map was drawn. The highest ASIRs in 2019 were observed in Denmark (224.9, 95% UI [194.6–257.5]), followed by Greenland (197.8, 95% UI [172.4–226.9]) and USA (193.9, 95% UI [174.5–215.5]) (Fig. 1a). The highest ASPRs were observed in Denmark (2702, 95% UI [2342–3069.7]), followed by Greenland (2252.3, 95% UI [1960.7–2570.8]) and USA (2245, 95% UI [1945.1–2546.4]) (Fig. 1b). Top 5 ASDRs were observed in Barbados (7.1, 95% UI [3.4–13.2]), Saint Kitts (5.1, 95% UI [2.4–9.9]), Hungary (4.9, 95% UI [1.9–10.4]), Saint Lucia (3.9, 95% UI [1.9–7.4]) and Russia (3.8, 95% UI [1.9–7.4]) (Fig. 1c). Similarly, Top 5 ASDALYRs were observed in Barbados (102, 95% UI [51.7–182.2]), Hungary (84.5, 95% UI [38.9–175.1]), Saint Kitts (74.7, 95% UI [38.1–138.8]), Russia (68.8, 95% UI [35.5–130]) and Ukraine (67, 95% UI [34.3–121.5]) (Fig. 1d). More details were shown in Supplementary Table 2 and Fig. 1a–d*.*

### Gender and age patterns of PAD-related disease burden

To reveal the patterns of gender and age in PAD-related disease burden, including incidence, prevalence, death, DALY, YLL and YLD and their ASRs by age and gender were analyzed. Notably, in 2019, females had a higher incidence (6.8 million, 95% UI [5.9–7.7]) and prevalence (76.1 million, 95% UI [66.6–86.2]) than males (3.7 million, 95% UI [3.2–4.3]; 37.3 million, 95% UI [32.5–42.6]). Fewer females died from PAD (36.3 thousand, 95% UI [15.8–74.8]) than males (37.7 thousand, 95% UI [17.6–79.5]). Females had 0.7 million (95% UI [0.4–1.4]) DALY while male had 0.8 million (95% UI [0.5–1.2]). Notably, male had higher YLL (0.6 million, 95% UI [0.3–1.2]) than female (0.4 million, 95% UI [0.2–0.9]), and less YLD (0.2 million, 95% UI [0.1–0.3]) than female (0.3 million, 95% UI [0.2–0.6]) (Supplementary Tables 1 and 3).

**Figure 2:**
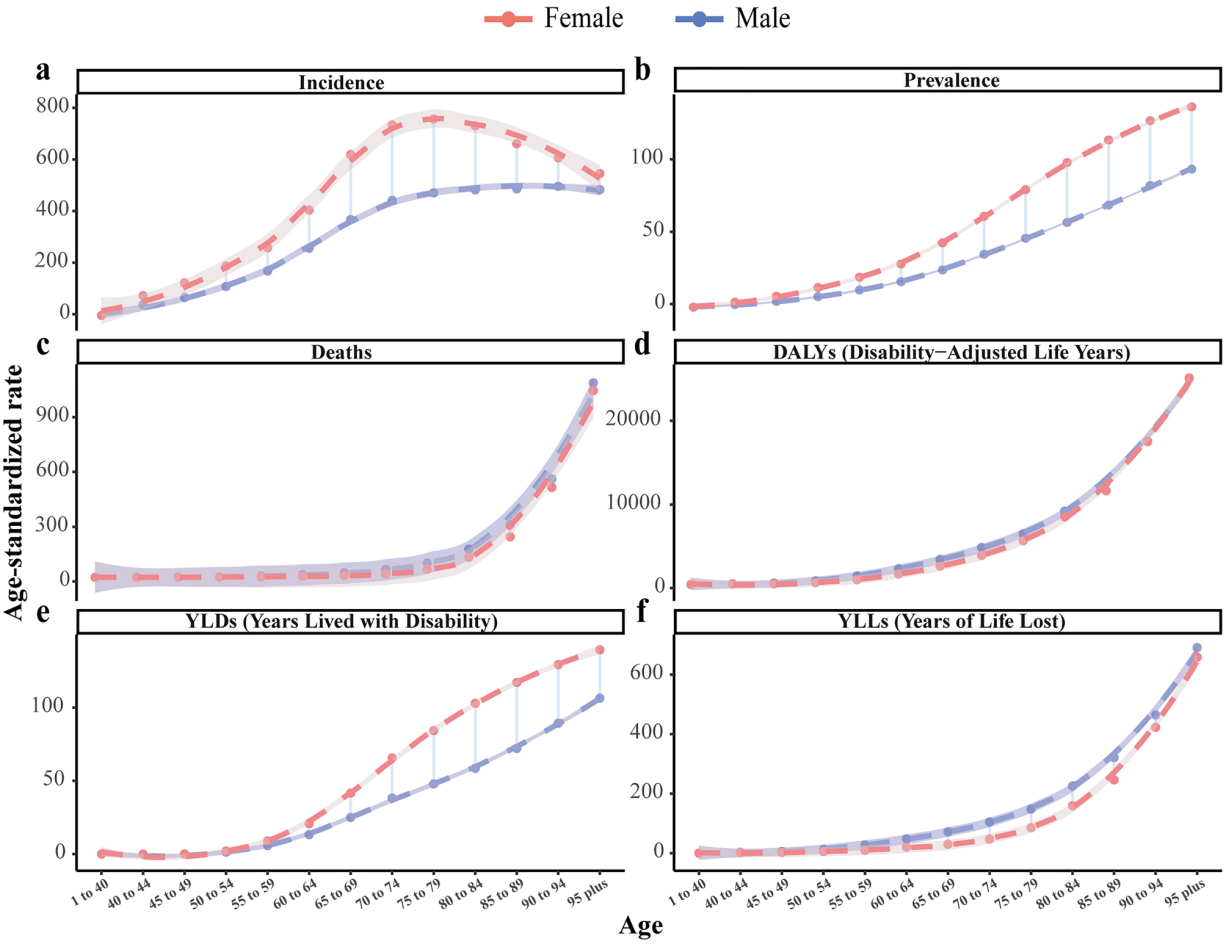
The differences between male and female in PAD-related ASIR (**a**), ASPR (**b**), ASDR (**c**), ASDALYR (**d**), ASYLDR (**e**), ASYLLR (**f**) in 2019 among different ages groups. ASIR: age-standardized incidence rate; ASPR: age-standardized prevalence rate; ASDR: age-standardized deaths rate; ASDALYR: age-standardized disability-adjusted life-years rate; ASYLDR: age-standardized years lived with disability rate; ASYLLR: age-standardized years of life lost rate; PAD: peripheral arterial disease.

Generally, ASIR and ASPR in both genders increased with age. Both ASIR and ASPR in females overwhelmed males. However, there was a moderate decline of ASIR in females over 75–79 y/o whereas ASIR gradually increased in males (Fig. 2a–b). Both genders shared the comparable trend of ASDR and ASDALY, increasing with age (Fig. 2c–d). Generally, males had more ASDR and ASDALYR than females in all age group. Females had higher ASYLDR in all age group compared to males while opposite situation was observed in ASYLLR (Fig. 2e–f).

### Trends of PAD-related burden

**Figure 3:**
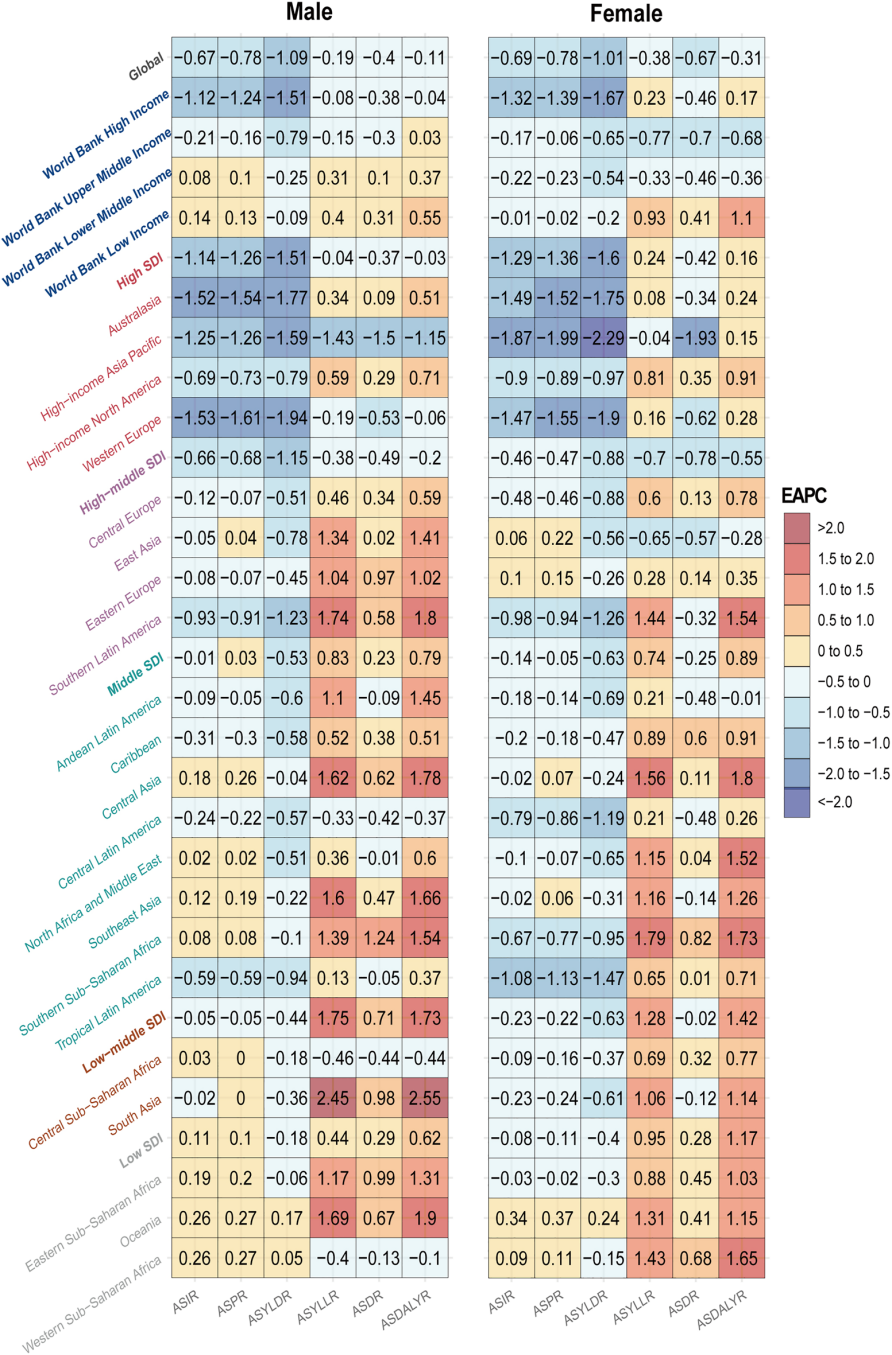
The EAPCs of PAD-related ASIR, ASPR, ASDR, ASDALYR, ASYLDR and ASYLLR in globe, SDI and WBI regions, by sex. The number indicates the corresponding EAPCs. Red indicates higher values, while blue indicates lower values of EAPCs. ASIR: age-standardized incidence rate; ASPR: age-standardized prevalence rate; ASDR: age-standardized deaths rate; ASDALYR: age-standardized disability-adjusted life-years rate; ASYLDR: age-standardized years lived with disability rate; ASYLLR: age-standardized years of life lost rate; PAD: peripheral arterial disease; EAPC: estimated annual percentage change; SDI: socio-demographic index; WBI: World Bank income level.

We calculated EAPCs separately by gender to assess the trends of PAD-related burden (ASDR, ASDALYR, ASPR and ASIR) (Fig. 3 and Supplementary Table 4).

Generally, the global ASIR showed a downward trend in both genders (male EAPC: − 0.67, 95% CI [− 0.72 to − 0.63]; female EAPC: − 0.69, 95% CI [− 0.73 to − 0.64]). High SDI regions exhibited a significant decline in ASIR for both genders (male EAPC: − 1.14, 95% CI [− 1.26 to − 1.01]; female EAPC: − 1.29, 95% CI [− 1.45 to − 1.14]), followed by high-middle SDI regions. Only males in low SDI regions showed an upward trend of ASIR (EAPC: 0.11, 95% CI [0.09–0.13]). ASPR also exhibited similar tendencies to ASIR in global level (EAPC: − 0.78, 95% CI [− 0.84 to − 0.72]). Significant downward trends of ASIR were observed in High-income Asia Pacific (EAPC: − 1.78, 95% CI [− 1.95 to − 1.61]), Australasia (EAPC: − 1.54, 95% CI [− 1.72 to − 1.36]), and Western Europe (EAPC: − 1.53, 95% CI [− 1.67 to − 1.38]). The significant downward trends of ASIR were observed in Japan (EAPC: − 1.77, 95% CI [− 1.96 to − 1.59]) and the United Kingdom (EAPC: − 1.82, 95% CI [− 2.05 to − 1.59]). Similar results were observed in ASPR, the top 2 downward trends were also observed in United Kingdom (EAPC: − 1.95, 95% CI [− 2.21 to − 1.69]) and Japan (EAPC: − 1.93, 95% CI [− 2.12 to − 1.74]).

Contrary to the global downward trend (EAPC: − 0.19, 95% CI [− 0.26 to − 0.12]), the ASDR showed upward trends in middle (EAPC: 0.86, 95% CI [0.82–0.91]), low-middle (EAPC: 1.57, 95% CI [1.54–1.6]), and low SDI regions (EAPC: 0.76, 95% CI [0.69–0.84]). The ASDALYR showed similar trends to ASDR. Regionally, the steepest declining trends in ASDR and ASDALYR were observed in high-income Asia Pacific (EAPC: − 0.46, 95% CI [− 0.54 to − 0.36] and EAPC: − 1.8, 95% CI [− 1.95 to − 1.64], respectively). South Asia (EAPC: 1.88, 95% CI [1.81–1.96]) and Central Asia (EAPC: 1.84, 95% CI [1.71–1.97]) exhibited the greatest increasing trends in ASDR. Southern Sub-Saharan Africa (EAPC: 1, 95% CI [0.8–1.19) and Eastern Sub-Saharan Africa (EAPC: 0.73, 95% CI [0.64–0.81) had the most obvious upward trends of ASDALYR. At the national level, Republic of Korea (EAPC: − 2.56, 95% CI [− 3.12 to − 2]), Slovenia (EAPC: − 1.81, 95% CI [− 2.13 to − 1.49]), and Greenland (EAPC: − 1.58, 95% CI [− 1.98 to − 1.18]) showed the greatest ASDR declining trends. Slovakia (EAPC: 2.99, 95% CI [2.65–3.32]), Equatorial Guinea (EAPC: 2.39, 95% CI [2.27–2.51]), and Serbia (EAPC: 2.31, 95% CI [1.89–2.74]) exhibited the strongest ASDALYR upward trends, whereas the Republic of Korea (EAPC: − 2.74, 95% CI [− 2.98 to − 2.5]), Greenland (EAPC: − 1.71, 95% CI [− 2. to − 1.42]), and Japan (EAPC: − 1.64, 95% CI [− 1.81 to − 1.46]) showed the most critical ASDALYR downward trends. More details of the EAPCs were showed in Supplementary Table 4.

### The correlation between EAPC and SDI

**Figure 4:**
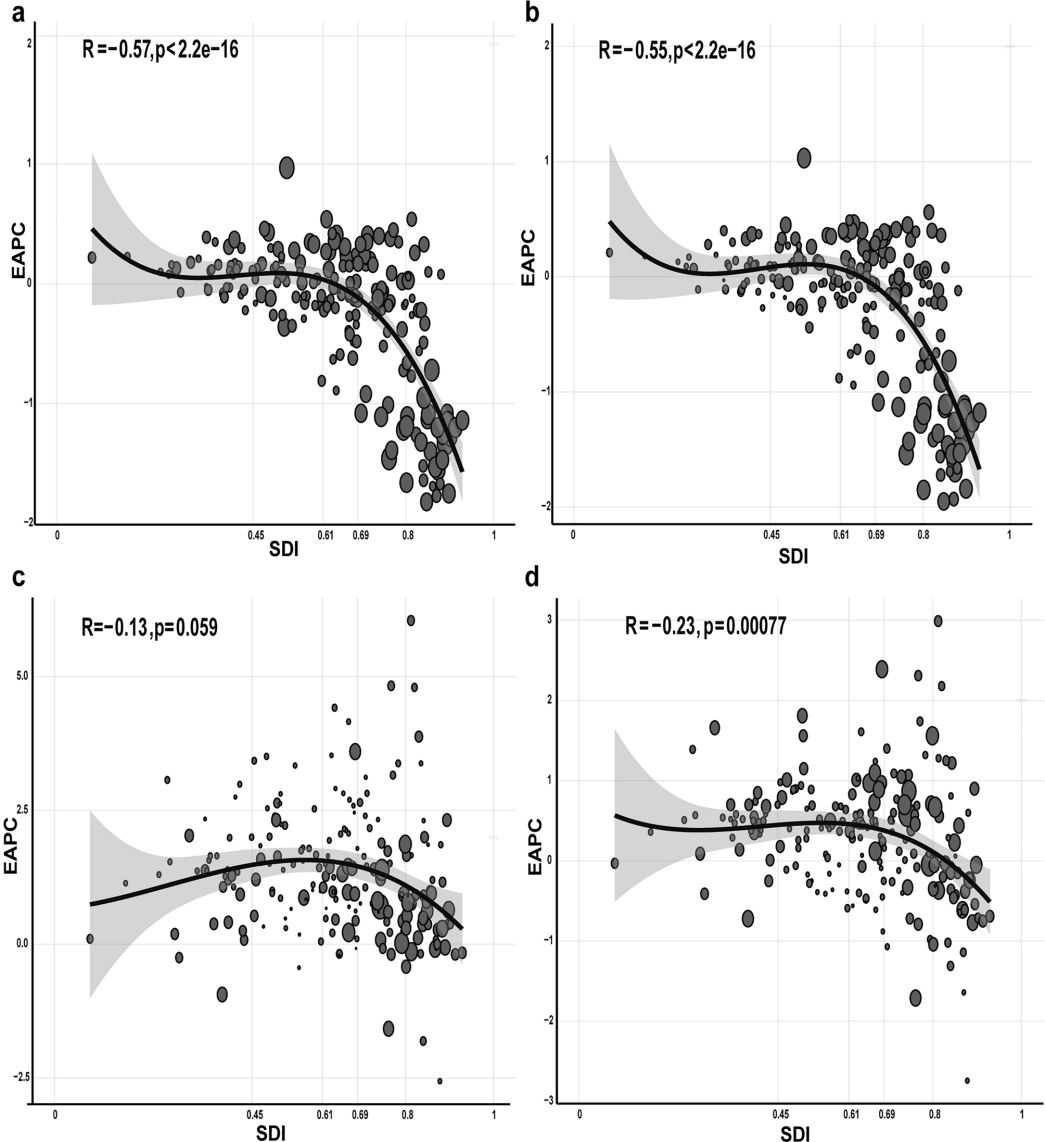
The correlation between the EAPCs and SDI. The size of the circle increased with the corresponding EAPCs number of ASIR (**a**), ASPR (**b**), ASDR (**c**), and ASDALYR (**d**). ASIR: age-standardized incidence rate; ASPR: age-standardized prevalence rate; ASDR: age-standardized deaths rate; ASDALYR: age-standardized disability-adjusted life-years rate; EAPC: estimated annual percentage change; SDI: socio-demographic index.

Overall, the EAPC of ASIR (R-sq = − 0.57; *P* < 0.001), ASPR (R-sq = − 0.55; *P* < 0.001) and ASDALYR (R-sq = − 0.23; *P* < 0.001) were negatively correlated with SDI level. The EAPCs of ASIR, ASPR and ASDALYR were higher in low-, low-middle and middle SDI regions, but dropped dramatically in high-middle and high SDI regions. It indicated that PAD-related burden in lower SDI regions could be substantially underestimated (Fig. 4).

### Contribution of attributable risk factors for PAD-related burden

To display PAD attributable risk factors and predict temporal trends, PAF of ASDR and ASDALYR was stratified by gender, SDI, and WBI regions to analyze individually (Supplementary Table 5).

The PAF of attributable risk factors varied significantly between male and female. In global level, smoking was the primary attributable risk factor for PAD-related burden in males (ASDR: 33.4%; ASDALYR: 43.4%; ASYLLR: 43%; ASYLDR: 44.9%) and high SBP was the primary attributable risk factor for females (ASDR: 25.3%; ASDALYR: 27.6%; ASYLLR: 26.5%; ASYLDR: 28.9%). As for ASDR, in 2019, the highest PAFs of smoking in males were in high-middle SDI regions (42.4%), upper-middle WBI regions (42.5%), Eastern Europe (54.3%), East Asia (49.6%), and Southeast Asia (46.1%). In 2019, the highest PAFs of high SBP for females were observed in high-middle SDI regions (26.4%), upper-middle WBI regions (27.3%), Western Sub-Saharan Africa (31.7%), Southern Sub-Saharan Africa (31.4%), and Southeast Asia (29.2%). The PAFs for these attributable risk factors for ASDALYR, ASYLLR, and ASYLDR in 2019 were also showed in Supplementary Table 5.

**Figure 5:**
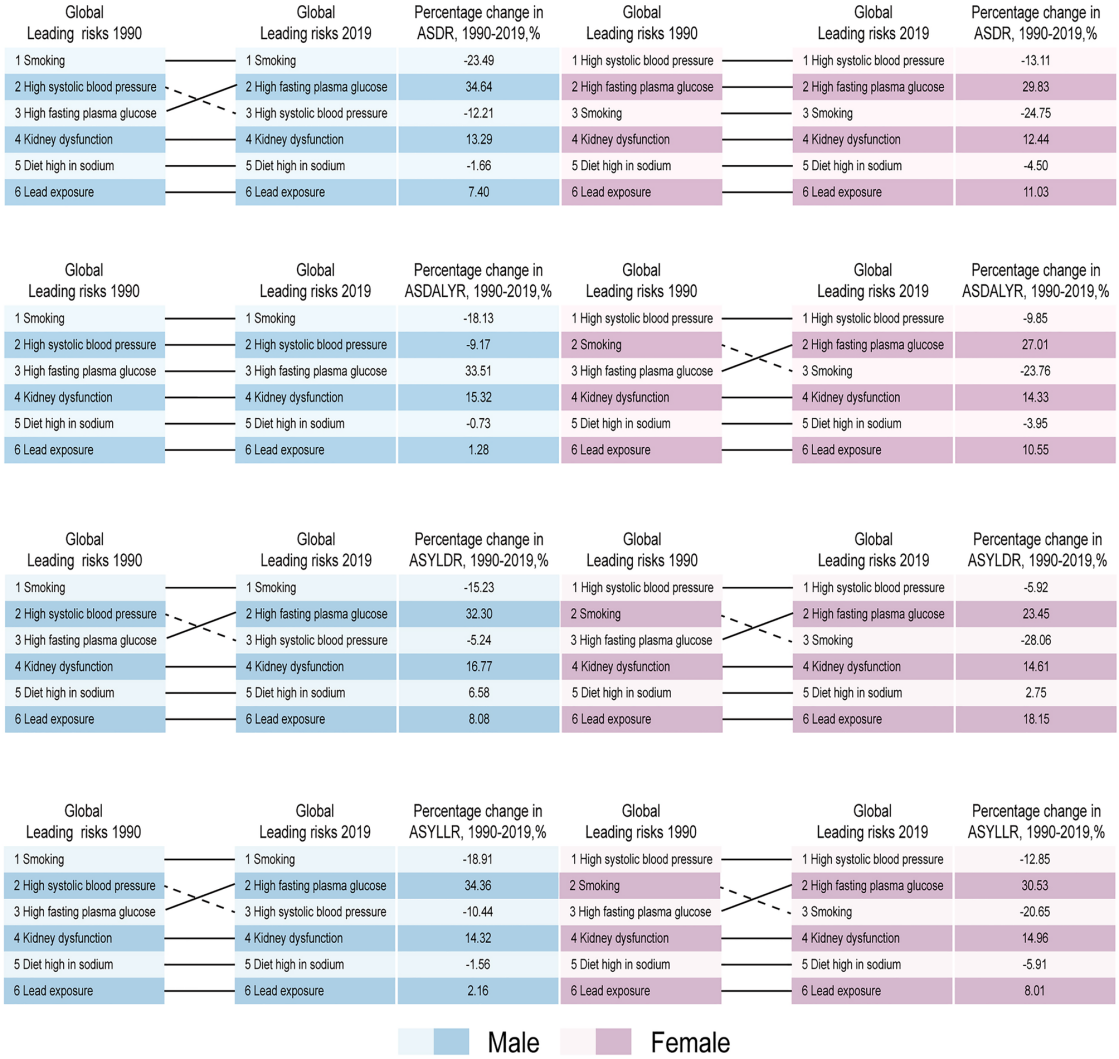
Rankings of attributable risk factors for PAD-related ASDR, ASDALYR, ASYLDR, ASYLLR and its percentage changes, by sex, 1990–2019. ASDR: age-standardized deaths rate; ASDALYR: age-standardized disability-adjusted life-years rate; ASYLDR: age-standardized years lived with disability rate; ASYLLR: age-standardized years of life lost rate; PAD: peripheral arterial disease.

The percentage change of these attributable risk factors to PAD-related burden from 1990 to 2019 were presented (Fig. 5 and Supplementary Table 5). As for ASDR in global level, smoking in males decreased by 23.49%, whereas high SBP in females decreased by 13.11%. Global ASDALYR exhibited similar tendencies. However, high FPG has become ranked 2 for ASDR, ASYLLR, and ASYLDR in males, and also grew dramatically to rank 2 for ASDR, ASDALYR, ASYLLR, and ASYLDR in females (Fig. 5). Notably, in high SDI regions, high FPG has become ranked 1 for ASDR in males whereas smoking remained to be ranked 1 for ASDALYR, ASYLLR, and ASYLDR. Furthermore, high FPG has been recognized as ranked 1 for ASDR, ASDALYR, ASYLLR in females (Supplementary Fig. 1a). However, in low SDI regions, high SBP seemed to be the predominant attributable risk factor for PAD-related burden in male and female, instead of smoking (Supplementary Fig. 1E). Regarding, the rank for PAFs of attributable risk factors to PAD-related burden from 1990 to 2019 in different SDI quintiles were detailed in Supplementary Fig. 1.

**Figure 6:**
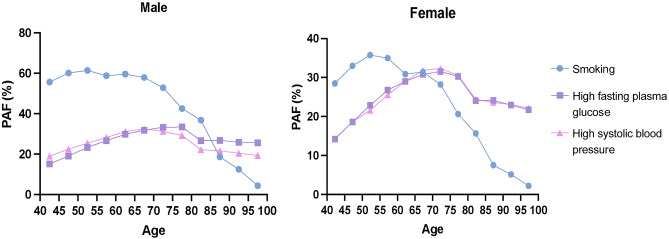
The PAFs of attributable risk factors for PAD-related ASDR in 2019 among different age groups, by sex. PAF: population attributable fraction.

Smoking, high SBP and high FPG were the primary risk factors for PAD-related burden. To clarify PAD-related burden caused by those attributable risk factors, we classified these risk factors according to different age groups and genders. As for males, smoking contributed to the most ASDR before 85–90 y/o, while high FPG contributed to the most ASDR after 85–90 y/o. In terms of females, smoking accounted for the most ASDR before 65–70 y/o, while high FPG and high SBP accounted for the most ASDR after 65–70 y/o (Fig. 6).

### Trend of attributable risks for PAD-related burden

**Figure 7:**
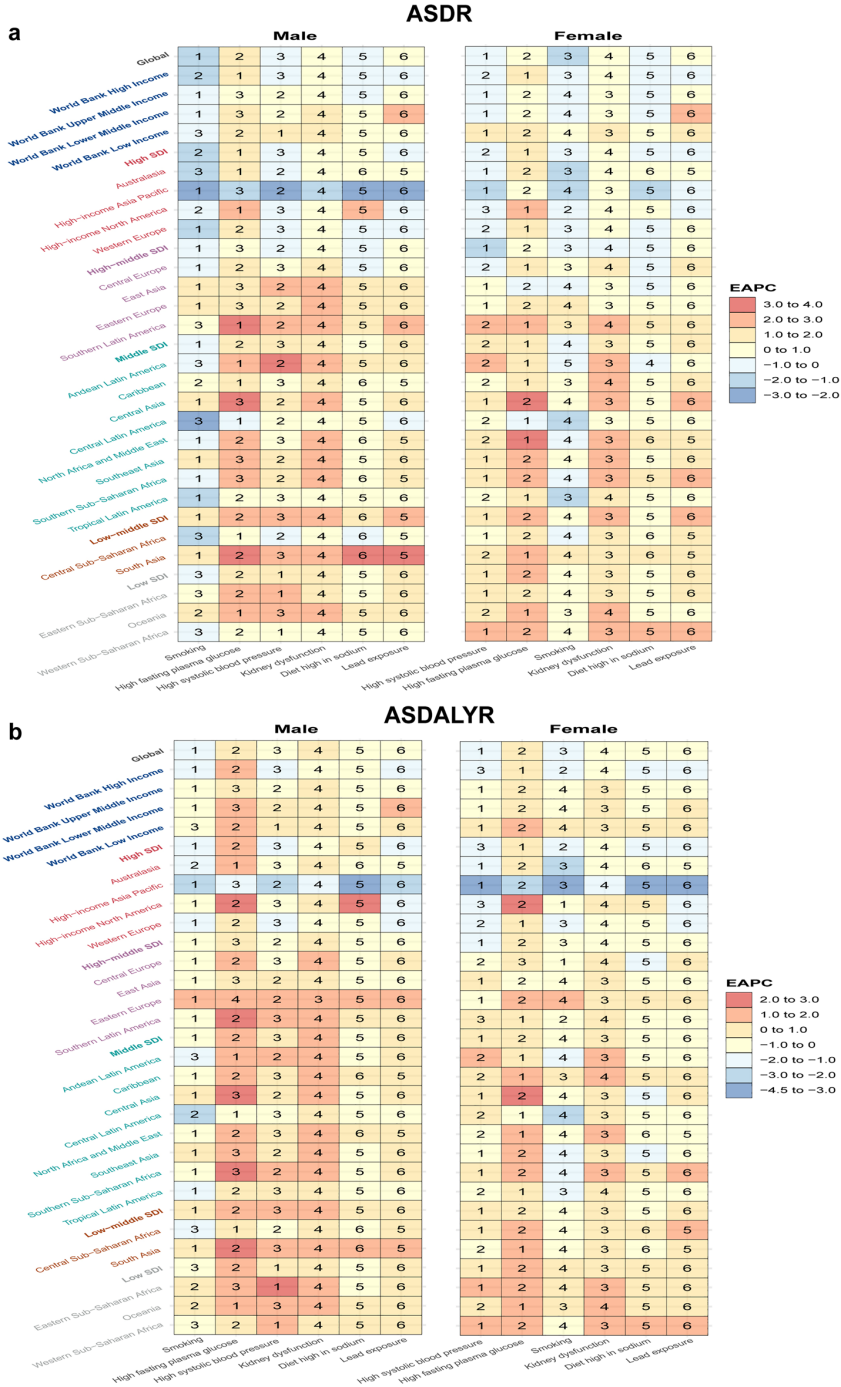
The EAPCs of attributable risk factors for PAD-related burden in globe, SDI, WBI and 21 GBD regions, by sex. Numbers show the ranking level (1 = highest, 6 = lowest) by the numbers of EAPC for corresponding ASDR (**a**) and ASDALYR (**b**). Red indicates higher values, while blue indicates lower values of EAPCs. ASDR: age-standardized deaths rate; ASDALYR: age-standardized disability-adjusted life-years rate; EAPC: estimated annual percentage change; SDI: socio-demographic index; WBI: World Bank income level; PAD: peripheral arterial disease.

EAPC was calculated to forecast the trends of these attributable risk factors related PAFs of ASDR and ASDALYR stratified by sex, SDI quintile and regions (Supplementary Table 5). Globally, the ASDR caused by smoking, high SBP and diet high in sodium showed downward trends (EAPCs: − 1, 95%CI [− 1.06 to − 0.94], − 0.79, 95%CI [− 0.89 to − 0.7] and − 0.22, 95%CI [− 0.27 to − 0.17]). The ASDR caused by high FPG, kidney dysfunction and lead exposure revealed upward trends with EAPCs by 1.07, 95%CI [0.97–1.18], 0.22, 95%CI [0.13–0.32] and 0.15, 95%CI [0–0.3]. Notably, ASDR caused by smoking showed a global decreasing trend in males (EAPC: − 1.02, (95%CI [− 1.05 to − 1.0]). The greatest declining trend was observed in high SDI regions by − 1.24, 95%CI [− 1.28 to − 1.21] while the ascending trend was found in low-middle SDI regions by 1, 95%CI [0.94–1.07]. High SBP related ASDR showed a downward trend globally in females by − 0.98, 95%CI [− 1.14 to − 0.82]. The greatest declining trend was observed in high-middle SDI regions by − 1.05, 95%CI [− 1.17 to − 0.93] whereas the highest ascending trend was found in low-middle SDI regions by 1.64, 95%CI [1.60–1.63] (Fig. 7a and Supplementary Table 5).

Globally, the ASDALYR caused by smoking, high SBP, diet high in sodium and lead exposure showed downward trends with EAPC by − 1.19% (95%CI [− 1.22 to − 1.16]), − 0.99% (95%CI [− 1.05 to − 0.92]), − 0.61% (95%CI [− 0.66 to − 0.55]) and − 0.32% (95%CI [− 0.44 to − 0.2]). The ASDALYR caused by high FPG showed an upward trend with EAPC by 0.59% (95%CI [0.52–0.66]). Notably, ASDALYR caused by smoking presented a declining trend with EAPC globally in males by − 1.11, 95%CI [− 1.13 to − 1.08]. Notably, the greatest declining trend was observed in high SDI region by − 1.35, 95%CI [− 1.39 to − 1.3] while the ascending trend was found in low-middle SDI region by 0.06, 95%CI [0.03–0.08]. High SBP related ASDALYR showed a downward trend with EAPC globally in females by − 1.15, 95%CI [− 1.23 to − 1.06]. The greatest declining trend was observed in high-middle SDI regions by 1.58, 95%CI [− 1.77 to − 1.4] whereas the highest ascending trend was found in low SDI regions by 0.44, 95%CI [0.37–0.51] (Fig. 7b *and* Supplementary Table 5).

Of note, in global level, high FPG showed a most dramatic growing tendency for ASDR by 1.07, 95%CI [0.97–1.18] and ASDALYR by 0.59, 95%CI [0.52–0.66] in male and female. Furthermore, high FPG related ASDR and ASDALYR were predicted to increase most rapidly in low-middle SDI regions (EAPC: 2.44, 95% CI [2.4–2.49] and EAPC: 1.32, 95% CI [1.28–1.35], respectively) (Fig. 7 and Supplementary Table 5).

## Discussion

The PAD-related disease burden has been underestimated over the past three decades and significant heterogeneity was found in terms of gender, age, socioeconomic and risk factor exposure. Although ASDR and ASDALYR showed a slight downward global trend, lower SDI regions are undergoing a rapid growth of PAD-related burden compared to higher SDI regions. Gender differences were observed in PAD-related burden and attributable risk factors. High SBP was the predominant risk factor for PAD-related ASDR and ASDALYR for females, whereas smoking was the primary risk factor for PAD-related ASDR and ASDALYR for males. High FPG has become the global notable risk factor with a remarkable upward trend for PAD-related ASDR and ASDALYR. Smoking caused more disease burden in male before 85–90 y/o and female before 65–70 y/o, while high FPG and high SBP caused more burden after that. Our results might serve as an imperative extension to the present studies and provide epidemiological data and potential tailored approaches for health care policymakers and social economists.

Similar to earlier global epidemiological findings of PAD based on GBD 2017 study, our study demonstrated an increase in incidence, prevalence, mortality, and DALY cases. However, the ASRs for these four metrics decreased during the past decades. Notably, a mild decrease in PAD-related burden was observed in ASDR compared to other ASCVDs, such as stroke with 34% reduction of ASDR^18^, and ischemic heart disease with 30.8% reduction of ASDR^19^. These results indicated that PAD-related death burden had been underestimated with poor management.

The global ASIR, ASPR, ASDR, and ASDALYR of PAD showed a declining trend. Our results indicated that the PAD-related disease burden decreased as economy developed^20,21^. Moreover, the EAPCs of these four metrics were negatively correlated with SDI levels, which were consistent with the previous studies^22–24^. The burden of evidence was suggestive that SDI has a significant impact on the burden of cardiovascular disease through non-health determinants of health^25,26^. Our findings suggested that people in lower SDI regions may be more vulnerable to PAD, which may be a result of less uptake of fruits and micronutrients^27^. According to current studies, deficiencies in these substances, such as fiber in grains and vitamins C, D, and E in fruits and vegetables, may increase the risk of cardiovascular disease including PAD^28,29^. Specific diets have shown positive effects in preventing PAD, such as the Mediterranean diet^30^. Social determinants of health are also crucial to PAD-related disease burden. Lower SDI regions have lower income, lower education levels, and less social support—each of which is associated with higher rates of PAD-related disease burden^31,32^. Additionally, the population in lower SDI regions has been exposed to more cardiovascular disease risks, such as smoking^33^, hypertension^34^ and diabetes^35^. Prevention, detection, and treatment of PAD, such as early screening and management of major risk factors will reduce PAD-related burden^36,37^. More tailored public health strategies should be adopted in low SDI regions, such as low-cost screening for PAD, management of risk factors and standardized treatment^38,39^.

We quantified the attributable burden and trends for PAD risk factors in the GBD database. In 2019, smoking, high SBP, and high FPG were the top 3 attributable risk factors for PAD-related ASDR and ASDALYR.

Diabetes was an independent risk factor for ASCVD, such as stroke, coronary heart disease, and PAD^40^. PAD patients with diabetes had a fivefold higher risk of amputation^41–43^. Our study showed that high FPG was a major contributor to ASDR and ASDALYR in PAD patients with a most significant upward trend. We also discovered that high FPG contributed to the highest ASDR after 85–90 y/o in males and caused a great amount of ASDR after 65–70 y/o in females. Additionally, high FPG was predicted to cause more PAD-related burden in lower SDI regions. This may be related to rising prevalence of diabetes in lower SDI regions^44^. Recent studies showed high-income countries had made substantial improvements in health care services, complication rates, and diabetes deaths^45^. Thus, diabetes management should be emphasized, especially in lower SDI regions and males aged over 85–90 y/o and females aged over 65–70 y/o.

Similar to diabetes, smoking is an important PAD risk factor. Smoking cessation is associated with decreased mortality and improved amputation-free survival among symptomatic PAD patients^46,47^. Our study found that smoking was the main contributor for PAD in males, consistent with previous reports^48–50^. Moreover, we also found that smoking contributed to the highest ASDR before 85–90 y/o in males and accounted for the highest ASDR before 65–70 y/o in females. Our study also indicated that the attribution of smoking to disease burden is declining globally, especially in high SDI and WBI regions. A study based on GBD 2019 found that higher SDI regions implemented better tobacco restriction policies, which reduced smoking prevalence and improved population health^51^. Therefore, the public and healthcare providers should strengthen smoking management, especially in lower SDI regions and males aged before 85–90 y/o and females before 65–70 y/o.

Blood pressure control reduced cardiovascular-related risks for death and other adverse outcomes. Several population-based studies have shown that hypertension is associated with PAD^52,53^. Our studies showed that high SBP contributed to the largest ASDR and ASDALYR of PAD in females and was the third risk factor of disease burden in males. We also found that high SBP contributed to the highest ASDR in females aged over 65–70 y/o. Though high SBP led to less disease burden globally, as observed in our study, it aggravated more disease burden of PAD in low, low-middle and middle SDI and low WBI regions compared to higher SDI and WBI regions, implying that more focus is needed on SBP control in females, especially aged over 65–70 y/o, and in lower income regions^54^.

Several studies have shown an association between chronic kidney disease and PAD^55,56^, Other studies reported poorer outcomes for PAD patients with chronic kidney disease, both in limb loss and mortality^57–59^. According to our results, kidney dysfunction was the fourth contributor to the PAD disease burden. It was supposed to cause more PAD-related burden, especially in lower SDI regions and lower WBI level countries. Statins and angiotensin-converting enzyme (ACE) inhibitors were reported to associate with improved renal function and reduced cardiovascular risk in patients with PAD^60^. Based on current evidence, initiation of appropriate treatment (e.g., statins and ACE inhibitors) should be implemented to preserve renal function and improve PAD-related morbidity and mortality^61^.

There are still limitations to consider when interpreting the study's findings. Firstly, our analysis depended on initial GBD studies' quality, lower SDI regions are less likely to have ankle-brachial index test, and other diagnostics approaches to PAD, which may lead to data omission. Secondly, GBD does not stratify PAD based on whether affected individuals were symptomatic or asymptomatic. Nor is it clear if PAD diagnostic criteria across countries were consistent. Thirdly, as with any observational study, there are likely unmeasured confounding factors outside this analysis' scope.

In conclusion, PAD as a public health challenge has been underestimated for three decades. Higher SDI regions are seeing a decline in PAD burden, but lower SDI regions are seeing a rise. High SBP was the leading cause of PAD-related death and DALYs for females, while smoking was the leading cause for males. High FPG has become the secondary critical risk factor predicted to cause more PAD disease burden, especially in lower SDI regions. These findings helped formulate tailored strategies to alleviate disease burden. Future research should focus on developing interventions targeting risk factors to reduce PAD-related burden.

### Supplementary Information

Supplementary Legends.

Supplementary Figure 1.

Supplementary Table 1.

Supplementary Table 2.

Supplementary Table 3.

Supplementary Table 4.

Supplementary Table 5.

## Data Availability

Data are available in a public, open access repository. See: https://ghdx.healthdata.org/gbd-results-tool.
