# Supplementary material for: Global disease burden and its attributable risk factors of peripheral arterial disease
Source: Sci Rep. 2023 Nov 14;13:19898. doi: 10.1038/s41598-023-47028-5 (PMC10645774; doi:10.1038/s41598-023-47028-5)
Supplement: Supplementary file 1 — Supplementary Legends. [file 41598_2023_47028_MOESM1_ESM.docx]

**Supplementary Figure 1.** **Rankings of attributable risk factors for PAD-related burden and its percentage change, by sex and SDI regions, 1990-2019.** Rankings of risk factors for PAD-related burden and its percentage change in high SDI (a), high-middle SDI (b), middle SDI (c), low-middle SDI (d) and low SDI (e) regions, by sex, 1990-2019. ASDR: age-standardized deaths rate; ASDALYR: age-standardized disability-adjusted life-years rate; ASYLDR: age-standardized years lived with disability rate; ASYLLR: age-standardized years of life lost rate; SDI: socio-demographic index.

**Supplementary Table 1: Incident cases, prevalence, deaths and DALYs of peripheral artery disease in globe, SDI and WBI regions and its fold change between 1990-2019, by sex.** UI: Uncertainty Intervals; SDI: socio-demographic index; WBI: World Bank income Level; DALYs: Disability-adjusted life-years.

**Supplementary Table 2. The ASIR, ASPR, ASDR and ASDALYR of PAD and its percentage change between 1990 and 2019, in globe, SDI, WBI, 21 GBD regions and 204 countries and territories, by sex.** ASIR: age-standardized incidence rate; ASPR: age-standardized prevalence rate; ASDR: age-standardized deaths rate; ASDALYR: age-standardized disability-adjusted life-years rate; UI: Uncertainty Intervals; SDI: socio-demographic index; WBI: World Bank income Level.

**Supplementary Table 3. Absolute number and age-standardized rate of YLLs and YLDs for PAD in globe, SDI and WBI regions and its percentage change between 1990-2019, by sex.** ASYLDR: age-standardized years lived with disability rate; ASYLLR: age-standardized years of life lost rate; YLLs: Years of life lost. YLDs: Years lived with disability; UI: Uncertainty Intervals; SDI: socio-demographic index; WBI: World Bank income Level.

**Supplementary Table 4. EAPCs of ASIR, ASPR, ASDR and ASDALYR in globe, SDI, WBI, 21 GBD regions and 204 countries and territories, by sex.** ASIR: age-standardized incidence rate; ASPR: age-standardized prevalence rate; ASDR: age-standardized deaths rate; ASDALYR: age-standardized disability-adjusted life-years rate; PAD: peripheral arterial disease; EAPC: estimated annual percentage change; SDI: socio-demographic index; WBI: World Bank income level; CI: confidential interval.

**Supplementary Table 5: Attributable risk factors' PAFs and its EAPCs for PAD-related burden in 1990 and 2019, in globe, SDI and WBI regions, by sex.** ASDR: age-standardized deaths rate; ASDALYR: age-standardized disability-adjusted life-years rate; ASYLDR: age-standardized years lived with disability rate; ASYLLR: age-standardized years of life lost rate; PAF: population attributable fraction; PAD: peripheral arterial disease; EAPC: estimated annual percentage change; SDI: socio-demographic index; WBI: World Bank income level; CI: confidential interval.
