# Supplementary material for: Global disease burden and its attributable risk factors of peripheral arterial disease
Source: Sci Rep. 2023 Nov 14;13:19898. doi: 10.1038/s41598-023-47028-5 (PMC10645774; doi:10.1038/s41598-023-47028-5)
Supplement: Supplementary file 2 — Supplementary Figure 1. [file 41598_2023_47028_MOESM2_ESM.docx]

**Disease burden and its attributable risk factors of peripheral arterial disease: findings from the global burden of disease study 2019**

Yayu You^1†^, Zhuo Wang^1, 2†^, Zhehui Yin^1^, Qinyi Bao^1^, Shuxin Lei^1^, Jiaye Yu^1^, Xiaojie Xie^1^*

^†^These authors have contributed equally to this work and share first authorship

^1^Department of Cardiology, Second Affiliated Hospital, Zhejiang University School of Medicine, Hangzhou, Zhejiang 310009 China

^2^International Institutes of Medicine, Fourth Affiliated Hospital, Zhejiang University School of Medicine, Yiwu, 322000 China

**Correspondence:**

Xiaojie Xie, MD, PhD.,Department of Cardiology,Second Affiliated Hospital, Zhejiang University School of Medicine,88 Jiefang Road, Hangzhou, Zhejiang 310009, China

Tel: (86)571-87784700

Fax: (86)571-87783777

E-mail: [xiexj@zju.edu.cn](mailto:xiexj@zju.edu.cn)


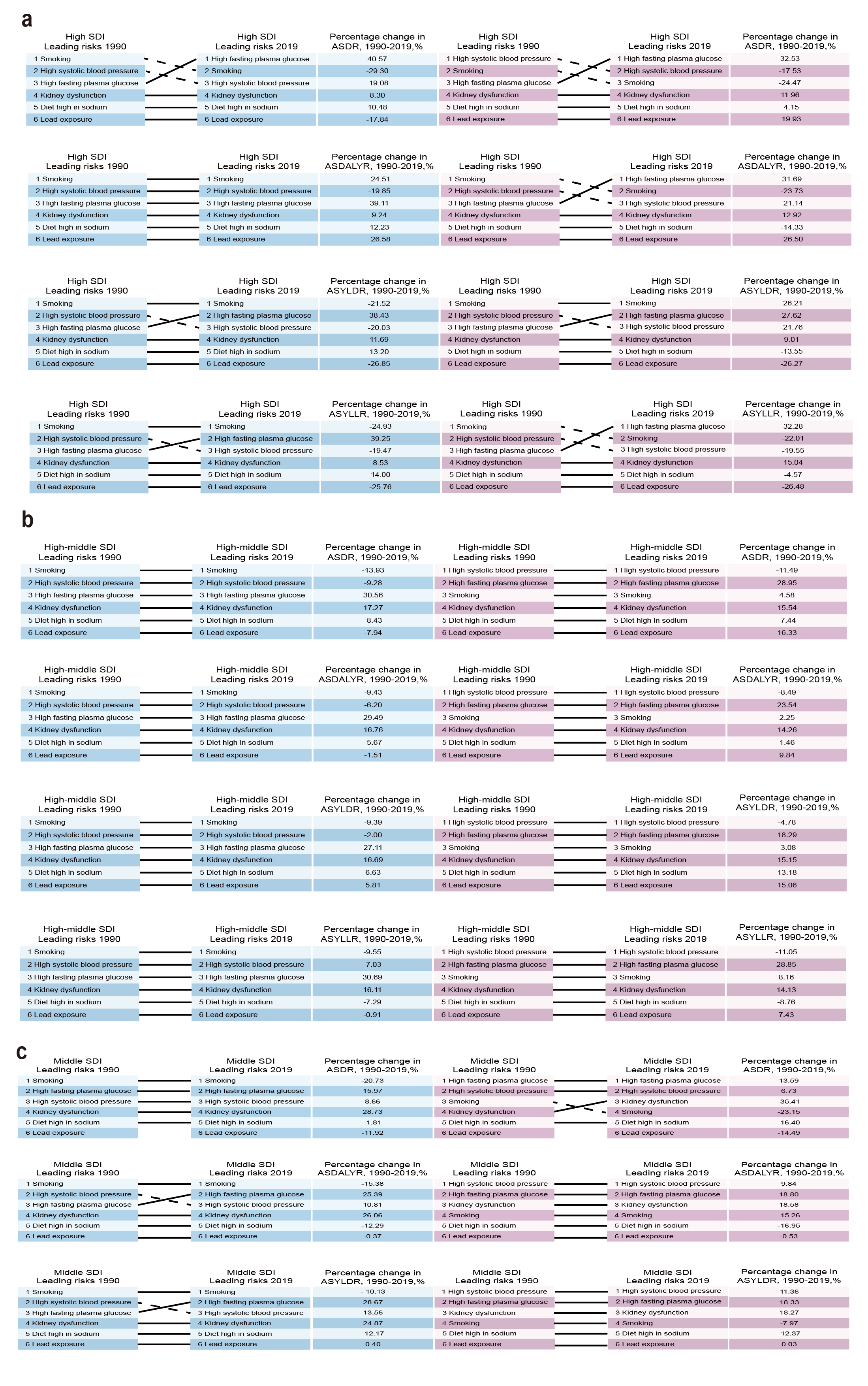

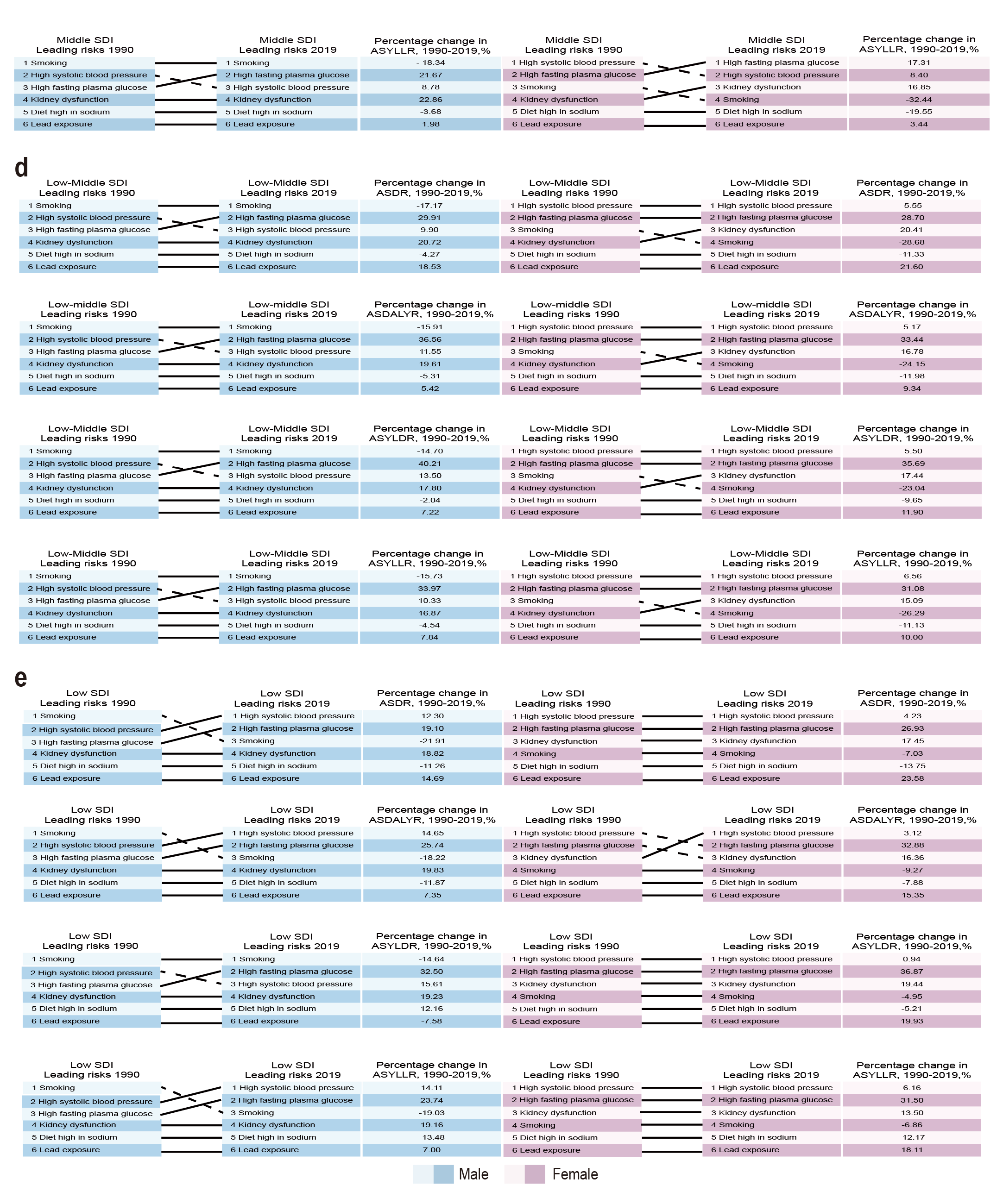


**Supplementary Figure 1.** **Rankings of attributable risk factors for PAD-related burden and its percentage change, by sex and SDI regions, 1990-2019.** Rankings of risk factors for PAD-related burden and its percentage change in high SDI (a), high-middle SDI (b), middle SDI (c), low-middle SDI (d) and low SDI (e) regions, by sex, 1990-2019. ASDR: age-standardized deaths rate; ASDALYR: age-standardized disability-adjusted life-years rate; ASYLDR: age-standardized years lived with disability rate; ASYLLR: age-standardized years of life lost rate; SDI: socio-demographic index.
